# Theoretical Study on the Complexes of Benzene with Isoelectronic Nitrogen-Containing Heterocycles

**DOI:** 10.1002/cphc.200700587

**Published:** 2008-04-03

**Authors:** Weizhou Wang, Pavel Hobza

**Affiliations:** [a] Institute of Organic Chemistry and Biochemistry, Academy of Sciences of the Czech Republic and Centre for Biomolecules and Complex Molecular SystemsFlemingovo nám. 2, 166 10 Prague 6, Czech Republic, Fax: (+420) 220-410-320 E-mail: pavel.hobza@uochb.cas.cz

**Keywords:** ab initio calculations, density functional calculations, dimer, nitrogen heterocycles, pi interactions

## Abstract

The π–π interactions between benzene and the aromatic nitrogen heterocycles pyridine, pyrimidine, 1,3,5-triazine, 1,2,3-triazine, 1,2,4,5-tetrazine, and 1,2,3,4,5-pentazine are systematically investigated. The T-shaped structures of all complexes studied exhibit a contraction of the C—H bond accompanied by a rather large blue shift (40–52 cm^−1^) of its stretching frequency, and they are almost isoenergetic with the corresponding displaced-parallel structures at reliable levels of theory. With increasing number of nitrogen atoms in the heterocycle, the geometries, frequencies, energies, percentage of s character at C, and the electron density in the C—H σ antibonding orbital of the complexes all increase or decrease systematically. Decomposition analysis of the total binding energy showed that for all the complexes, the dispersion energy is the dominant attractive contribution, and a rather large attraction originating from electrostatic contribution is compensated by its exchange counterpart.

## 1. Introduction

Interactions between π systems have been the focus of attention for a long time because they play key roles in many fields such as supramolecular chemistry, drug design, biochemistry, crystal engineering, and many other new cross-disciplines associated with molecular science.^1^–^4^ As a prototype of π–π interactions, the benzene dimer has been studied both theoretically and experimentally,^5^–^8^ and these studies have greatly improved our understanding of the fundamental physics of π–π interactions. In many instances, however, not only the aromatic benzene ring but also N-containing heterocycles are involved in π–π interactions, such as π–π stacking in metal complexes with aromatic nitrogen-containing ligands^9^ and nucleobase stacking in nucleic acids,^4^ three nucleobases of which, namely, cytosine, thymine, and uracil, are pyrimidine derivatives. It is therefore important to systematically study π–π interactions between benzene and aromatic nitrogen heterocycles.

**Figure 1:**
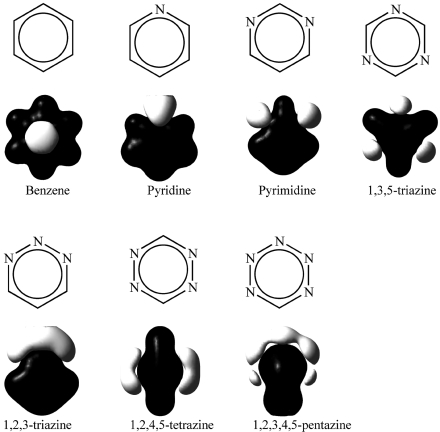
MP2/aug-cc-pVDZ molecular electrostatic potential for benzene and isoelectronic nitrogen-containing heterocycles. The black region represents the positive part of the electrostatic potential, and the white region the negative part of the electrostatic potential.

The very electronegative heteroatom nitrogen withdraws electron density from the aromatic heterocycle. With increasing number of nitrogen atoms in the ring, the π electron density in the ring decreases and the molecular electrostatic potential (MEP) becomes more positive. As can be seen in Figure 1, the MEP value is negative above the center of the benzene ring but positive in the aromatic nitrogen heterocycles pyridine, pyrimidine, 1,3,5-triazine, 1,2,3-triazine, 1,2,4,5-tetrazine, and 1,2,3,4,5-pentazine. The negative MEP of benzene is responsible for the existence of the T-shaped benzene dimer. The absence of the negative MEP above the center in the other compounds suggests that their dimers might not be T-shaped like the benzene dimer. Furthermore, lower π-electron density in the ring means less π-electron repulsion between π-donor and π-acceptor ring systems. Hence, the interactions between the benzene molecule and pyridine, pyrimidine, 1,3,5-triazine, 1,2,3-triazine, 1,2,4,5-tetrazine, and 1,2,3,4,5-pentazine should be stronger than that in the benzene dimer. Ugozzoli and Massera investigated the intermolecular potential of the benzene⋅⋅⋅1,3,5-triazine complex in detail.^10^ In contrast to the results of Tsuzuki et al. for the benzene dimer, calculated at the same level of theory,^11^ the interaction between benzene and 1,3,5-triazine is indeed much stronger. Note that this can be at least partially ascribed to more attractive dispersion energy, since the polarizability of 1,3,5-triazine is higher than that of benzene. Geerlings et al. ascertained that substituted benzenes with electron-withdrawing or electron-donating groups bind more strongly to pyridine than unsubstituted benzene,^12^–^13^ and this is also inconsistent with the Hunter–Sanders rules, which state that the electron-donating groups will destabilize the π–π interaction.^1^ The calculations mentioned mainly focus on the different structures and energies of only one complex rather than studying the interactions of benzene with different aromatic nitrogen-containing heterocycles systematically. Several interesting questions regarding the complexes formed by benzene with different aromatic nitrogen-containing heterocycles remain unanswered: 1) relative stability of T-shaped and parallel-displaced structures; 2) spectral shift of the C—H stretching frequency on complex formation (the T-shaped benzene dimer exhibits a blue shift of the C—H stretching frequency);^14^, ^15^ and 3) decomposition of the interaction energy. The last point is very important because it can help us further understand the nature of the π–π interaction in the complexes.

**Figure 2:**
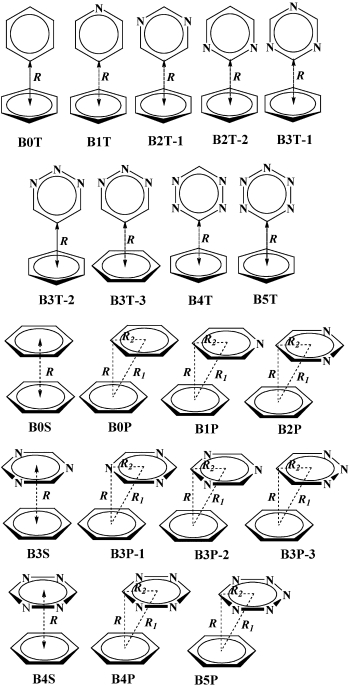
Selected configurations of the complexes formed by benzene with isoelectronic nitrogen-containing heterocyclic compounds.

To answer the above questions, we select six complexes formed between benzene and its isoelectronic nitrogen-containing heterocycles. Both T-shaped and stacked structures are considered (Figure 2). Note that our aim is not the evaluation of accurate absolute interaction energies but merely the relative values for different complexes. For comparison, the T-shaped and stacked structures of the benzene dimer are also included.

## Computational Methods

The π–π interactions in the considered complexes are mostly governed by London dispersion forces, theoretical determination of which is quite difficult. Second-order Møller–Plesset theory (MP2) has been shown to be effective and accurate in determining the equilibrium structures and binding energies of many hydrogen-bonded complexes. However, previous ab initio studies indicated that MP2 tends to overestimate binding in the case of π–π stacking interactions.^11^, ^16^–^20^ The π–π stacking interactions are properly described only if a higher level of theory, such as the coupled-cluster method with singles, doubles, and noniterative triples [CCSD(T)] is adopted. CCSD(T) calculations with extended basis sets are, however, quite demanding. Fortunately, in their study on the relative energies of the multiply substituted benzene dimers and benzene dimer, Sinnokrot and Sherrill found that the MP2/aug-cc-pVDZ method yields relative energies very similar to the more expensive CCSD(T)/aug-cc-pVTZ method.^21^ Evidently, this is due to a compensation of errors. Since the main aim of the present work is to compare the binding energies of the various dimers, the MP2/aug-cc-pVDZ method was used for most calculations.

The reliability of the MP2/aug-cc-pVDZ method for the present dimers was demonstrated by performing high-level calculations for the benzene⋅⋅⋅1,3,5-triazine complex. The CCSD(T) binding energy at the complete basis set (CBS) was approximated as the sum of the MP2/CBS interaction energy and the CCSD(T) correction term. The CCSD(T) correction term, ΔCCSD(T), is defined in the present paper as the difference between CCSD(T) and MP2 binding energies in the aug-cc-pVDZ basis set. The MP2/CBS binding energy was estimated by extrapolation of the calculated binding energies with the aug-cc-pVXZ basis sets by using a fitting of the form *a*+*b* exp(−*cX*), where *X*=2, 3, and 4 for aug-cc-pVDZ, aug-cc-pVTZ, and aug-cc-pVQZ, respectively.^22^, ^23^ All the structures of the benzene⋅⋅⋅1,3,5-triazine complex were fully optimized at the MP2/aug-cc-pVDZ and MP2/aug-cc-pVTZ theory levels. The MP2/aug-cc-pVQZ calculations use the MP2/aug-cc-pVTZ optimized geometries.

The basis set superposition error (BSSE) of the binding energy was systematically eliminated by using the standard counterpoise (CP) correction method of Boys and Bernard.^24^ The frozen-core approximation was applied throughout.

The total interaction energies 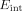 were decomposed into first-order 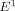, second-order 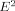, and higher order [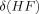] terms by using the DFT–SAPT perturbation treatment.^25^–^29^ 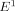 is the sum of the electrostatic interaction energy 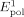 and the first-order exchange energy 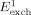. 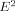 denotes the sum of the second-order induction energy 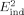 and the second-order dispersion energy 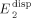. Details of the DFT–SAPT method can be found elsewhere.^25^–^29^ The DFT–SAPT calculations were performed with the MP2/aug-cc-pVDZ geometries. The PBE0AC exchange–correlation functional with density fitting and aug-cc-pVDZ basis set was used systematically. The basis set dependency of the individual interaction energy terms has been studied by Jansen et al.,^28^ who found that the aug-cc-pVDZ basis set is a reasonable compromise between computational cost and accuracy; this basis set used provided accurate energy terms with the exception of the dispersion energy, which was underestimated by about 10–20 %. In the PBE0AC exchange–correlation potential, the incorrect asymptotic behavior of the corresponding PBE exchange potential has been corrected by the gradient-regulated asymptotic correction approach of Grüning et al.^30^ This approach needs a shift parameter which approximates the difference between the vertical ionization potential and the negative HOMO energy obtained from the respective standard Kohn–Sham calculation. Herein the ionization potentials and the HOMO values were calculated at the PBE0/aug-cc-pVDZ level. The calculated shift parameters were 0.0715, 0.0765, 0.0775, 0.0801, 0.0815, 0.0846, and 0.0876 a.u. for benzene, pyridine, pyrimidine, 1,3,5-triazine, 1,2,3-triazine, 1,2,4,5-tetrazine, and 1,2,3,4,5-pentazine, respectively.

The Gaussian03 suite of programs^31^ was used for the ab initio molecular orbital calculations to evaluate the geometries, harmonic frequencies, and the total interaction energies. The natural bond orbital (NBO) analysis^32^ was carried out by using the MP2-optimized structure, the MP2 electron density, and the built-in subroutine of the Gaussian03 program suite. The total interaction energy (DFT–SAPT procedure) was decomposed by using the MOLPRO 2006.1 software package.^33^

## 2. Results and Discussion

### 2.1. Binding Energies of Benzene⋅⋅⋅1,3,5-Triazine Complex

Table 1 summarizes the binding energies for the T-shaped (B3T-1), sandwich (B3S), and parallel-displaced (B3P-1, B3P-2) configurations of the benzene⋅⋅⋅1,3,5-triazine complex at various levels of theory.

**Table 1:** CP-corrected binding energies [kcal mol^−1^] for different configurations of benzene⋅⋅⋅1,3,5-triazine complex.[a]

	MP2 aug-cc-pVDZ	MP2 aug-cc-pVTZ	MP2 aug-cc-pVQZ	MP2 CBS	ΔCCSD(T) aug-cc-pVDZ	CCSD(T) CBS
B3T-1	−3.23	−3.80	−3.95	−4.00	0.96	−3.04
B3S	−3.97	−4.64	−4.82	−4.89	2.16	−2.73
B3P-1	−4.75	−5.56	−5.78	−5.86	2.64	−3.22
B3P-2	−4.60	−5.40	−5.61	−5.69	2.64	−3.05

[a] See Figure 2 and for the structures and computational details.

First, the MP2 level is discussed. Evidently, passing from aug-cc-pVDZ to the much larger aug-cc-pVQZ basis set leads to a significant increase in binding energy. By extrapolation, we obtain the MP2/CBS binding energies, which are even larger than the aug-cc-pVQZ values, by up to 0.08 kcal mol^−1^. At the MP2/CBS level, the parallel-displaced structures are most stable, followed by sandwich and T-shaped ones. As expected, the CCSD(T) correction term is small for the T-shaped structure and considerably larger for sandwich and parallel-displaced structures. This term is systematically repulsive (by 2–5 kcal mol^−1^) for stacking structures of various DNA base pairs and amino acid pairs.^34^ By adding this term to the MP2/CBS one, we obtain the CCSD(T)/CBS binding energy (Table 1, last column). Evidently, these binding energies are smaller than the corresponding MP2 ones. Moreover, energy differences between these structures become smaller; at the MP2 level it is 1.9 kcal mol^−1^, while at the CCSD(T) level it is only 0.5 kcal mol^−1^. The parallel-displaced structure B3P-1 remains the global minimum, but the T-shaped structure is energetically close and the difference is smaller than 0.2 kcal mol^−1^. The sandwich structure B3S is now the least stable structure.

Table 1 shows that the binding energy of the T-shaped structure B3T-1 determined at the MP2/aug-cc-pVDZ level of theory is −3.23 kcal mol^−1^, which is close to the CCSD(T)/CBS binding energy of −3.04 kcal mol^−1^. Similarly, the binding energies for the T-shaped structure of the benzene dimer are −2.86 and −2.74 kcal mol^−1^ at the MP2/aug-cc-pVDZ and CCSD(T)/CBS levels of theory, respectively.^35^ The differences between the binding energies of the T-shaped structures of the benzene dimers and the benzene⋅⋅⋅1,3,5-triazine complex at the CCSD(T)/CBS and MP2/aug-cc-pVDZ levels are thus similar (0.3 and 0.37 kcal mol^−1^). This allows us to use the much cheaper MP2/aug-cc-pVDZ method for determining relative binding energies of the T-shaped structures of the complexes formed between benzene and its isoelectronic nitrogen-containing heterocycles. The MP2/aug-cc-pVDZ binding energies of the sandwich and parallel-displaced structures of the benzene⋅⋅⋅1,3,5-triazine complex differ from the CCSD(T)/CBS values by up to 1.6 kcal mol^−1^. The differences become more pronounced when the larger aug-cc-pVTZ and aug-cc-pVQZ basis sets are used. However, the MP2/aug-cc-pVDZ binding energies of the B3P-1 and B3P-2 structures differ by less than 0.2 kcal mol^−1^, which is very close to the CCSD(T)/CBS value (0.2 kcal mol^−1^). This indicates that the relative binding energies of the particular structural type are predicted at the MP2/aug-cc-pVDZ level of theory fairly accurately. This conclusion is in agreement with the finding of Sherrill et al. for the benzene dimer.^21^

Table 1 further shows that the CCSD(T)/CBS binding energies are almost identical for the T-shaped structure B3T-1 and the parallel-displaced structure B3P-2. Additionally, the binding energies of the T-shaped, sandwich, and parallel-displaced structures are systematically larger than the corresponding energies for the benzene dimer.^36^ Interestingly, the binding energy of B3P-1 is larger than that of B3P-2 at all levels of theory, which contradicts our chemical intuition. The electron lone pairs of one nitrogen atom in B3P-1 are above the benzene ring, and consequently stronger electron-electron repulsion is expected. This question is resolved in Section 2.3 by a detailed energy decomposition analysis.

### 2.2. Structure, Harmonic Vibrational Frequency, Binding Energy, and NBO Analyses of Benzene⋅⋅⋅X (X=Benzene, Pyridine, Pyrimidine, 1,3,5-Triazine, 1,2,3-Triazine, 1,2,4,5-Tetrazine and 1,2,3,4,5-Pentazine) Complexes

Table 2 lists selected geometrical parameters, shifts in C—H stretching frequency, changes in NBO electron density, derivatives of the permanent dipole moment, and binding energies of the complexes formed by benzene and its isoelectronic nitrogen-containing heterocycles, calculated at the MP2/aug-cc-pVDZ level. Unless otherwise noted, the C—H bonds discussed are those involved in the C—H⋅⋅⋅π hydrogen bonds in the T-shaped dimers. For the definition of *R*, *R*_1_, and *R*_2_, see Figure 2.

**Table 2:** Geometries [Å], harmonic vibrational frequencies [cm^−1^], changes in NBO electron density [e^−^], permanent dipole moment derivatives [D Å^−1^], and binding energies [kcal mol^−1^] of the complexes of benzene with isoelectronic nitrogen-containing heterocycles at the MP2/aug-cc-pVDZ level.[a]

	*r*_C−H_ (Monomer)	*r*_C−H_ (Dimer)	Δ*r*_C−H_	Δ 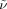 (C—H)	*R*	d*μ*/d*r*_C—H_	ΔED/CT	Δ%s-char	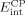
B0T	1.0944	1.0905	−0.0039	+41	3.3359	−0.42	30/196	+1.34 %	−2.86
B1T	1.0939	1.0904	−0.0035	+52	3.3346	−0.35	32/190	+1.38 %	−3.29
B2T-1	1.0927	1.0896	−0.0032	+41	3.3328	−0.23	33/185	+1.46 %	−3.84
B2T-2	1.0935	1.0902	−0.0032	+51	3.3286	+0.44	2/179	+1.51 %	−2.73
B3T-1	1.0934	1.0904	−0.0030	+48	3.2937	−0.38	6/181	+1.65 %	−3.22
B3T-2	1.0926	1.0894	−0.0032	+40	3.2772	−0.11			−4.49
B3T-3	1.0926	1.0894	−0.0032	+40	3.2782	−0.11			−4.49
B4T	1.0920	1.0894	−0.0025	+41	3.2517	−0.02	21/193	+1.74 %	−4.09
B5T	1.0920	1.0894	−0.0026	+42	3.2263	+0.09	23/215	+1.86 %	−4.88
			*R*_1_	*R*_2_	*R*				
B0S					3.5397				−2.54
B3S					3.3858				−3.97
B4S					3.3055				−5.08
B0P			3.5635	1.5366	3.2152				−3.88
B1P			3.4980	1.3579	3.2237				−4.52
B2P			3.4164	1.3305	3.1467				−5.23
B3P-1			3.3877	1.3178	3.1209				−4.74
B3P-2			3.4305	1.4275	3.1194				−4.60
B3P-3			3.3546	1.2249	3.1229				−6.46
B4P			3.3428	1.4638	3.0053				−6.11
B5P			3.2602	1.3550	2.9653				−7.42

[a] Δ %s-char is the percentage change in s character in a carbon hybrid orbital in the C—H bond; ΔED is the change in electron density in the C—H σ antibonding orbital, and CT the total charge transfer between the electron donor (benzene) and the electron acceptor (N heterocycle); d*μ*/d*r*_C—H_ is the derivative of the permanent dipole moment of the proton donor. Cartesian co-ordinates of all the stationary point geometries are given in the Supporting Information.

The structure and geometry of the complexes are discussed first. The distance between subsystems in the T-shaped, sandwich, and parallel-displaced structures decreases with increasing number of nitrogen atoms. The decrease is the smallest in the T-shaped structures (0.1096 Å). A larger decrease is found for sandwich structures (0.2342 Å), and the largest occurs for parallel-displaced structures (*R*_1_ decreases by 0.3033 Å). The shortening of the intermolecular distance is connected with an increase in binding energy. For the T-shaped, sandwich, and parallel-displaced structures, it is 2.02, 2.54 and 3.54 kcal mol^−1^, respectively. According to the positions of the nitrogen hetero atoms, the T-shaped dimers can be categorized into two groups: a) B0T, B1T, B2T-1, B3T-2, and B3T-3, and b) B2T-2, B3T-1, B4T, and B5T. In group a, there is no nitrogen atom to either side of the C—H bond involved in the C—H⋅⋅⋅π interaction, while in group b, two nitrogen atoms are near the C—H bond. Evidently, the binding energy increases with increasing number of nitrogen atoms in both groups. Note that the binding energy and geometry of B3T-2 are almost the same as those of B3T-3, which indicates free rotation about the *C*_6_ axis of benzene. Similarly, the parallel-displaced dimers can be categorized into three groups based on the number of nitrogen atoms above the benzene ring. Group a′ includes B0P, B1P, B2P, and B3P-3, in which no nitrogen atom is above the benzene ring. Group b′ includes only B3P-1, in which one nitrogen atom is above the benzene ring. The remaining structures are included in group c′, which has two nitrogen atoms above the benzene ring. The binding energy in all three groups increases with increasing number of nitrogen atoms.

In the case of T-shaped complexes, the binding energy of B2T-1 is larger than the binding energy of B2T-2, and the binding energy of B3T-2 is larger than that of B3T-1, whereas for parallel-displaced complexes, the binding energy of B3P-3 is larger than that of B3P-1, and that of B3P-2 is the lowest one among the three complexes. It is possible to conclude that binding energy increases with increasing distance of the nitrogen atoms from the benzene ring.

The geometry and spectral shift of the C—H bond in the T-shaped dimers were also investigated. The *C*_2*v*_ T-shaped structure of the benzene dimer was the first system for which the existence of a blue-shifting hydrogen bond was predicted.^14^, ^15^ Since then various types of blue-shifting hydrogen bonds have been confirmed experimentally, not only in the gas phase, but also in liquid and solid phases. The C—H⋅⋅⋅π blue-shifting hydrogen bond in the benzene dimer was, however, not found experimentally, and the experimental study pointed to a small red shift of the C—H stretching frequency on benzene dimerization.^8^ In a recent study, we suggested an explanation for this finding (global minimum *C_s_* T-shaped structure has a red shift, while a *C*_2*v*_ T-shaped structure, which is only a transition structure, has a blue shift).^37^ Table 2 shows that all C—H bond lengths in the T-shaped complexes are systematically contracted on complex formation. Contraction of the C—H bond is accompanied by an increase in C—H stretching frequency, that is, by a blue shift. However, as pointed out by McDowell and Buckingham,^38^ there is no correlation between the change in bond length and the shift in vibrational frequency. The largest C—H bond contraction was found for the benzene dimer, and the smallest for the benzene⋅⋅⋅1,2,4,5-tetrazine complex; the blue shift is, however, identical for both dimers. The largest blue shifts (52 and 51 cm^−1^) were found in benzene⋅⋅⋅pyridine and benzene⋅⋅⋅pyrimidine complexes.

The explanation of blue-shifting H-bonding is, unlike the case of red-shifting H-bonding, not unambiguous and several possibilities should be investigated. The first concerns the electrostatic theory of H-bonding and is based on the fact that elongation of the X—H bond of the proton donor is mostly connected with an increase of the proton-donor dipole moment. A larger dipole moment yields a larger dipole–dipole attraction, which is the reason for the elongation of the X—H bond and the subsequent red shift of the X—H stretching frequency. However, a few systems exhibit the opposite, that is, elongation of the X—H bond is connected with a decrease in dipole moment. In other words, contraction of the X—H bond results in an increase in the dipole moment of the proton donor. Analogously to the previous case, a larger dipole moment yields stronger dipole–dipole attraction, which is the reason for the contraction of the X—H bond and the subsequent blue shift of the X—H stretching frequency. The dipole-moment derivatives for the proton donors considered have mostly negative values (Table 2), consistent with bond contraction and a blue shift, with the exception of pyrimidine with the C—H proton donor located between two nitrogen atoms in the B2T-2 structure and 1,2,3,4,5-pentazine. The large positive value found for the pyrimidine monomer indicates elongation and red shifting of the C—H stretching vibration on dimerization. However, examining Table 2 reveals the opposite: the C—H bond in the B2T-2 dimer is contracted and is associated with the second largest blue shift. Evidently, in most cases the C—H spectral shift is explained by dipole-moment derivatives, but there are some exceptions. The second possibility is based on the theory developed by Alabugin et al., who proposed that the X—H bond length in the X—H⋅⋅⋅Y H-bond is controlled by a subtle balance of hyperconjugation and rehybridization.^39^ Hyperconjugation (electron-density shift from lone pairs of the proton acceptor to the σ* antibonding X—H orbital) results in elongation of the X—H bond and consequently a red shift of the X—H stretching frequency. Rehybridization, on the other hand, strengthens the X—H bond and results in a blue shift of the X—H stretching frequency. Both effects act simultaneously, and the final effect depends on the prevalent role of either contribution. Table 2 lists the values of the total charge transfer (CT) between the proton acceptor and the proton donor as well as the increase in electron density (ΔED) in the σ* antibonding orbital of a C—H bond. Evidently, the total CT is much larger than ΔED, which indicates a very small or negligible effect of hyperconjugation on the C—H bond length. In the case of the red-shifting H-bonding, the ΔED and CT values are practically identical. The changes in percentage s character (Δ%s-char) in the carbon hybrid orbital forming the C—H bond of the proton donor are systematically positive, that is, the s character increases on complex formation. This increase is connected with strengthening of the C—H bond, which is followed by an increase of the C—H stretching frequency. We found no correlation of the Δ%s-char values with the contraction of the C—H bond or the blue shift. Evidently, various factors are responsible for the magnitude of the blue shift and, among the three factors investigated, only rehybridization fully agrees with the spectral shift of benzene and nitrogen-containing heterocyclic compounds on complex formation.

### 2.3. Decomposition Analysis of the Total Binding Energy

Table 3 shows the SAPT decomposition of the total interaction energies for all the complexes. They are ordered according to the aforementioned categories. The electrostatic term 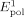 is systematically stabilizing for all the complexes studied. In each group of complexes, the absolute value of the electrostatic term increases with increasing number of nitrogen atoms. A larger value of this term correlates with the more positive molecular electrostatic potential of the heterocyclic compound with more nitrogen atoms. The first-order interaction energy (the sum of electrostatic and exchange-repulsion terms, 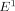 in Table 3) is, however, systematically repulsive due to a rather large repulsion value of the exchange-repulsion term. This term also increases with increasing number of nitrogen atoms, with the exception of B0T in group a of the T-shaped dimers. Note that lower π-electron density in the ring does not mean smaller π-electron repulsion between the aromatic rings. It is the intermolecular distance that plays the important role for the exchange-repulsion term.

**Table 3:** DFT–SAPT decomposition of interaction energy [kcal mol^−1^] for the complexes formed by benzene with isoelectronic nitrogen-containing heterocycles. Interaction energies at the MP2/aug-cc-pVDZ level are also listed here for comparison.

		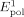	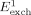	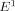	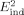	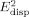	*E*^2^	δ(HF)	*E*_int_	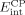 (MP2)
	B0T	−3.18	7.93	4.75	−0.34	−5.55	−5.89	−0.68	−1.82	−2.86
	B1T	−3.51	7.65	4.14	−0.43	−5.39	−5.82	−0.74	−2.42	−3.29
Group a	B2T−1	−3.85	7.71	3.87	−0.50	−5.33	−5.83	−0.74	−2.70	−3.84
	B3T−2	−4.73	8.63	3.91	−0.76	−5.57	−6.34	−0.95	−3.38	−4.49
	B3T−3	−4.73	8.64	3.91	−0.77	−5.57	−6.34	−0.95	−3.38	−4.49
	B2T−2	−3.04	7.32	4.29	−0.41	−5.10	−5.52	−0.70	−1.93	−2.73
Group b	B3T−1	−3.72	7.88	4.15	−0.54	−5.22	−5.77	−0.85	−2.46 (−2.27)[a]	−3.22
	B4T	−4.50	8.70	4.20	−0.77	−5.47	−6.23	−0.99	−3.02	−4.09
	B5T	−5.23	9.04	3.81	−1.05	−5.50	−6.54	−1.18	−3.92	−4.88
	B0S	−1.54	8.70	7.16	−0.17	−7.32	−7.49	−0.24	−0.57	−2.54
	B3S	−3.71	9.64	5.94	−0.19	−7.53	−7.72	−0.36	−2.15 (1.81)[a]	−3.97
	B4S	−4.79	10.96	6.17	−0.33	−7.93	−8.25	−0.37	−2.45	−5.08
	B0P	−4.55	14.08	9.53	−0.28	−9.49	−9.76	−1.19	−1.42	−3.88
Group a′	B1P	−5.32	14.33	9.02	−0.26	−9.56	−9.82	−1.25	−2.05	−4.52
	B2P	−6.46	15.42	8.95	−0.32	−9.88	−10.20	−1.41	−2.66	−5.23
	B3P−3	−7.94	16.92	8.98	−0.61	−10.26	−10.86	−1.69	−3.57	−6.46
Group b′	B3P−1	−5.67	14.17	8.50	−0.38	−9.29	−9.67	−1.20	−2.37 (−2.11)[a]	−4.74
	B3P−2	−5.30	13.29	7.99	−0.31	−8.95	−9.26	−0.92	−2.19 (−1.96)[a]	−4.60
Group c′	B4P	−7.20	15.88	8.68	−0.56	−9.73	−10.28	−1.42	−3.02	−6.11
	B5P	−8.85	17.34	8.48	−0.88	−10.07	−10.95	−1.60	−4.07	−7.42

[a] Values in parentheses are interaction energies at the CCSD(T)/aug-cc-pVDZ level of theory.

The second-order induction energy is much smaller than other energy components and increases with increasing number of nitrogen atoms. This term is least favorable for the sandwich structures and most favorable for the T-shaped structures. Following expectations, the second-order dispersion term is large and also increases with increasing number of nitrogen atoms with the exception of B0T and B1T in group a of the T-shaped dimers. The dispersion energy is largest for parallel-displaced structures followed by sandwich structures. The dispersion energy is systematically larger than the corresponding electrostatic term. The second-order interaction energies (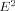 in Table 3) are attractive for all dimers, and their major part originates in dispersion energy.

When all energy terms [including δ(HF)] are put together, we obtain the total binding energy 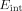. Table 3 shows that the order of the SAPT binding energies in each group is the same as that of the CP-corrected MP2/aug-cc-pVDZ binding energies. For the T-shaped dimers, the difference between the two binding energies is about 1 kcal mol^−1^, and the agreement will be more satisfactory if the larger aug-cc-pVTZ and aug-cc-pVQZ basis sets are used for SAPT calculations.^28^ However, for the sandwich and parallel-displaced dimers, the MP2/aug-cc-pVDZ binding energies differ from the SAPT values by up to 3.0 kcal mol^−1^, which is very similar to the case of the benzene dimer.^20^ The SAPT binding energies are very close to the corresponding CCSD(T)/aug-cc-pVDZ values (Table 3). Clearly, the SAPT binding energy is more accurate and the difference between the two energies is clearly due to the repulsive CCSD(T) correction term, which is missing in the MP2 binding energy but is at least partly included in SAPT binding energy. The SAPT binding energies of B2T-1, B4T, and B5T are almost the same as those of B2P, B4P, and B5P. By comparing the respective energy component of B3P-1 with that of B3P-2, we ascertained that the exchange-repulsion term of B3P-1 is indeed larger than that of B3P-2, which is consistent with our chemical intuition. However, the attractive induction, dispersion, and high-order terms of B3P-1 are all larger than those of B3P-2, which makes B3P-1 bind slightly more strongly than B3P-2.

## 3. Conclusions

We have performed MP2 and DFT–SAPT calculations to study the π–π interactions between benzene and the aromatic nitrogen heterocycles pyridine, pyrimidine, 1,3,5-triazine, 1,2,3-triazine, 1,2,4,5-tetrazine, and 1,2,3,4,5-pentazine. By analyzing the geometries, vibrational frequencies, natural bond orbitals, and binding energies, we conclude with the following remarks:

1. The C—H bond length in the T-shaped dimer is contracted on complex formation. This contraction is accompanied by a large blue shift of the C—H stretching frequency (between 40 and 52 cm^−1^). The dipole-moment derivatives and change in electron density in the σ* antibonding orbital of the C—H bond mostly correlate with the blue shift, while the change of rehybridization of the carbon atom in the C—H bond upon dimer formation always coincides with a blue shift.
2. The CCSD(T)/CBS binding energies are almost the same for T-shaped complex B3T-1 and parallel-displaced complex B3P-2. Also, the SAPT binding energies of B2T-1, B4T, and B5T are almost the same as those of B2P, B4P, and B5P. The results indicate that the T-shaped structure of each complex studied here is isoenergetic with the corresponding parallel-displaced structure, which is very similar to the case for benzene dimer.^6^
3. With increasing number of nitrogen atoms in the heterocycles, the distance between the rings decreases while the binding energy increases. For the T-shaped complexes, the percentage of s character at C and the electron density in the C—H σ* antibonding orbital and their change on complex formation increase with decreasing distance between the rings. Correspondingly, the change in C—H bond length decreases with decreasing intermolecular distance.
4. For all complexes investigated, the dispersion energy is the dominant attractive contribution. A rather large attraction originating from electrostatic contribution is compensated by its exchange counterpart. Generally, all energy components increase with increasing number of nitrogen atoms in the heterocycle.
